# Cell mediated ECM-degradation as an emerging tool for anti-fibrotic strategy

**DOI:** 10.1186/s13619-023-00172-9

**Published:** 2023-09-01

**Authors:** Peng Zhao, Tian Sun, Cheng Lyu, Kaini Liang, Yanan Du

**Affiliations:** Department of Biomedical Engineering, School of Medicine, Tsinghua-Peking Center for Life Sciences, Tsinghua University, Beijing, 100084 China

**Keywords:** Fibrosis, Extracellular matrix, Degradation, Matrix metalloproteinases

## Abstract

Investigation into the role of cells with respect to extracellular matrix (ECM) remodeling is still in its infancy. Particularly, ECM degradation is an indispensable process during the recovery from fibrosis. Cells with ECM degradation ability due to the secretion of various matrix metalloproteinases (MMPs) have emerged as novel contributors to the treatment of fibrotic diseases. In this review, we focus on the ECM degradation ability of cells associated with the repertoire of MMPs that facilitate the attenuation of fibrosis through the inhibition of ECM deposition. Besides, innovative approaches to engineering and characterizing cells with degradation ability, as well as elucidating the mechanism of the ECM degradation, are also illustrated. Studies conducted to date on the use of cell-based degradation for therapeutic purposes to combat fibrosis are summarized. Finally, we discuss the therapeutic potential of cells with high degradation ability, hoping to bridge the gap between benchside research and bedside applications in treating fibrotic diseases.

## Background

Fibrosis originates from an excessive deposition of the ECM caused by different types of tissue injuries, thus leading to organ dysfunction (Panizo et al. 2021). During the healing and regeneration process, fibroblasts are activated and differentiated into myofibroblasts, which then secrete ECM components to repair the tissue (Krafts 2010). The process of regeneration is accompanied by the restructuring of the ECM, primarily through the action of proteases that break down the ECM. Upon completion of regeneration, activated fibroblasts either undergo apoptosis (Micallef et al. 2012) or return to a quiescent state (Henderson et al. 2020). However, in fibrosis, fibroblasts remain activated and an abundance of ECM is produced. An imbalance between ECM synthesis and degradation leads to collagen deposition (Zhang and Shaw 2013), while the shifting of the balance between MMPs and tissue inhibitors of metalloproteinases (TIMPs) further exacerbates the process of fibrosis (Arthur 2003). MMP-mediated ECM degradation is immensely associated with cell migration, cell invasion (Cheng et al. 2010) and tissue remodeling (Green and Lund 2005). Briefly, bioactive ECM fragments generated from proteolytic degradation of the ECM promote the regulation of inflammation and tissue regeneration during the process of fibrosis (de Castro Bras and Frangogiannis 2020).

**Fig. 1:**
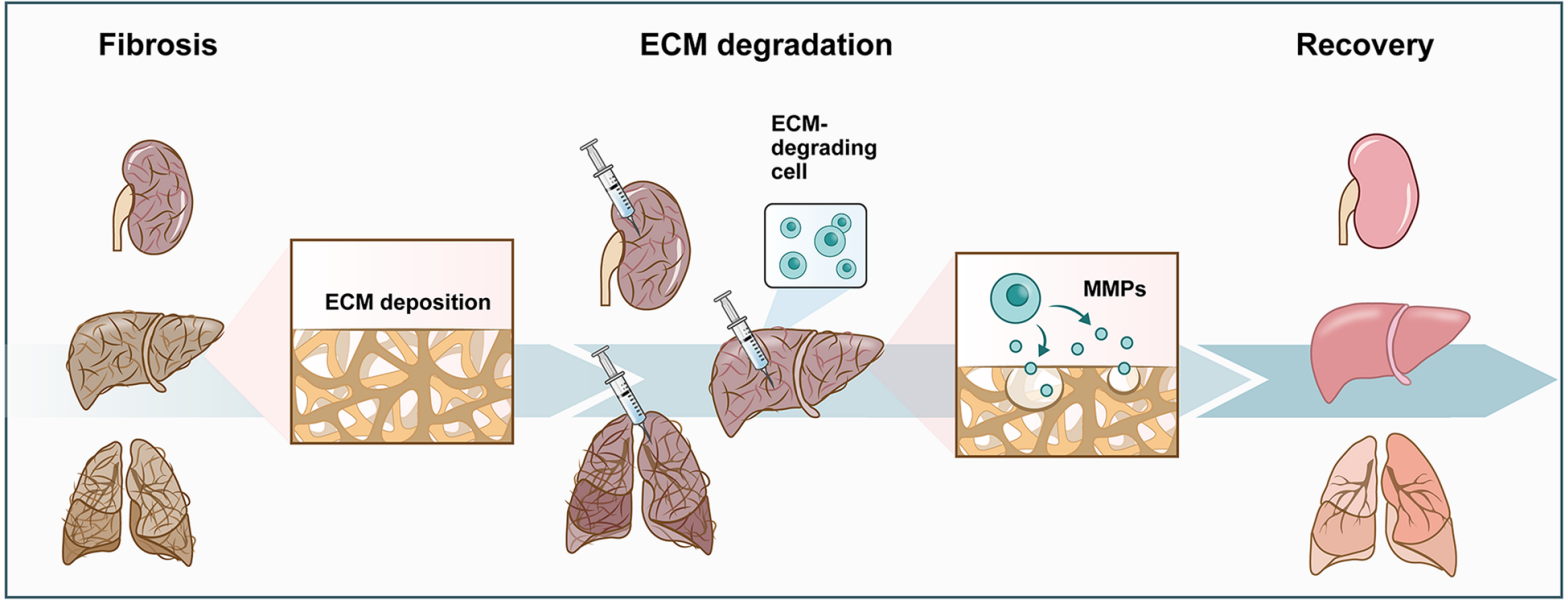
Schematic of ECM-degrading cell therapy applied to fibrosis. During fibrosis, the ECM is excessively secreted and deposited. Delivery of ECM-degrading cells to fibrotic organs or tissues can degrade the deposited ECM mainly by secreting MMPs, which are expected to  reduce excessive fibrosis

However, previous studies have paid inadequate attention to the ability of cells to degrade the ECM, which emerges as a potent property in the treatment of fibrotic diseases in the future. In this review, we introduce the potential cells with strong degradation abilities and prospect their applications in the treatment of fibrosis (Fig. 1).

## ECM degradation

### Proteases in ECM degradation

#### MMPs and their inhibitors

Proteases are comprised of serine protease, cysteine protease, aspartic protease and metalloproteinase, among which MMPs play a major role in the degradation of the ECM. To date, 28 members of the MMP family have been found in vertebrates, and at least 23 members have been detected in humans (Xi and Khalil 2017). The structure of MMPs from N-terminal to C-terminal generally includes (1) a hydrophobic signal peptide; (2) an N-terminal propeptide, which is cleaved by exogenous enzymes to activate the MMP zymogen; (3) a catalytic (CAT) domain with a zinc ion binding site that can catalyze the hydrolysis of peptide bonds; (4) a linker (hinge) region; and (5) a C-terminal hemopexin-like (HPX) domain, which recognizes its substrates. In addition, transmembrane-type MMPs contain a transmembrane (TM) domain and a cytoplasmic tail that regulates intracellular trafficking and activity, while membrane-type MMPs (MT-MMPs) are anchored to the cell membrane by glycosylphosphatidylinositol (Sagi and Gaffney 2015)*.*

According to substrate specificities, MMPs can be divided into collagenases, gelatinases, stromelysins, and matrilysins. Collagenases (e.g., collagenase 1 [MMP1], collagenase 2, neutrophil collagenase [MMP8], collagenase 3 [MMP13], and MT1-MMP) are dominant enzymes that degrade collagen fibers and unwind the triple helix. Meanwhile, gelatinases (e.g., gelatinase A [MMP2], gelatinase B [MMP9]) insert three fibronectin type II-like (FN2) repeats in the CAT domain to degrade gelatin (i.e., a product of the partial hydrolysis of collagen) and type IV collagen. Stromelysins (e.g., MMP3, MMP10, and MMP13) degrade a multitude of ECM components such as proteoglycans, laminin, fibronectin, gelatin, type IV and type IX collagen, while matrilysins (e.g., MMP7 and MMP26) degrade gelatin, fibronectin and type IV collagen but not the triple helical collagen (Sagi and Gaffney 2015). In addition, MMPs can also catalyze various non-ECM proteins. For instance, MMP7 catalyzes the hydrolysis of cytokines, growth factors, and receptors (Li et al. 2002).

The activity of MMPs is tightly regulated at the transcriptional level and by the activation of precursor zymogens and TIMPs. TIMPs bind to the CAT sites of MMPs in a substrate-like manner, thus inhibiting the degradation of substrates. Four homologous TIMPs (e.g., TIMP1, TIMP2, TIMP3, and TIMP4) can inhibit a variety of MMPs with divergent inhibitory effects. For example, the ability of TIMP1 and TIMP2 to bind to MMP3 is ten times stronger than their binding to MMP10 (Batra et al. 2012).

#### Plasmin

Plasmin is a proteolytic enzyme that specifically degrades fibrin in the body and is critical for the dissolution of clots. Plasminogen is the inactive precursor of plasmin that can be activated by two plasminogen activator (PA) systems: the tissue plasminogen activator (tPA) and the urokinase plasminogen activator (uPA) (Mahmood et al. 2018). Besides fibrin, plasmin has been reported to degrade several basement membrane proteins, including laminin and fibronectin, thus demonstrating its ability to degrade the ECM (Nakagami et al. 2000; Uemura et al. 2005). In addition, plasmin activates a variety of MMP zymogens, including MMP1, MMP2, MMP3, MMP9, MMP13, and MMP14, indicating that plasmin can indirectly degrade the ECM by activating MMPs (Deryugina and Quigley 2016).

#### Cathepsins

Cathepsins contain 15 members, which are located in the lysosome or secreted extracellularly. They can be classified by catalytic site residues as serine cathepsins (cathepsins A and G), cysteine cathepsins (cathepsins B, C, F, H, K, L, O, S, V, X, and W), and aspartate cathepsins (cathepsins D and E) (Kryczka and Boncela 2017). Some members of the cathepsin family have been found to degrade the ECM proteins. For instance, cysteines B, X, S, L, and H, which can catalyze type I collagen, type IV collagen, fibronectin, and laminin, are involved in ECM degradation in podosomes formed by fibroblasts (Tu et al. 2008) and macrophages (Jevnikar et al. 2012). Besides, cathepsin K is highly expressed in osteoclasts and shows potent collagenase activity, which plays an important role in cancer and bone resorption (Qian et al. 2022). Elastin, the main ECM fiber component that provides tissue elasticity, is known to be cleaved by cathepsins K, S, and V (Yoo et al. 2022). Moreover, cathepsin L has been found to activate pro-urokinase-type plasminogen activator (pro-uPA) (Tagirasa and Yoo 2022).

### Mechanism of ECM degradation

**Fig. 2:**
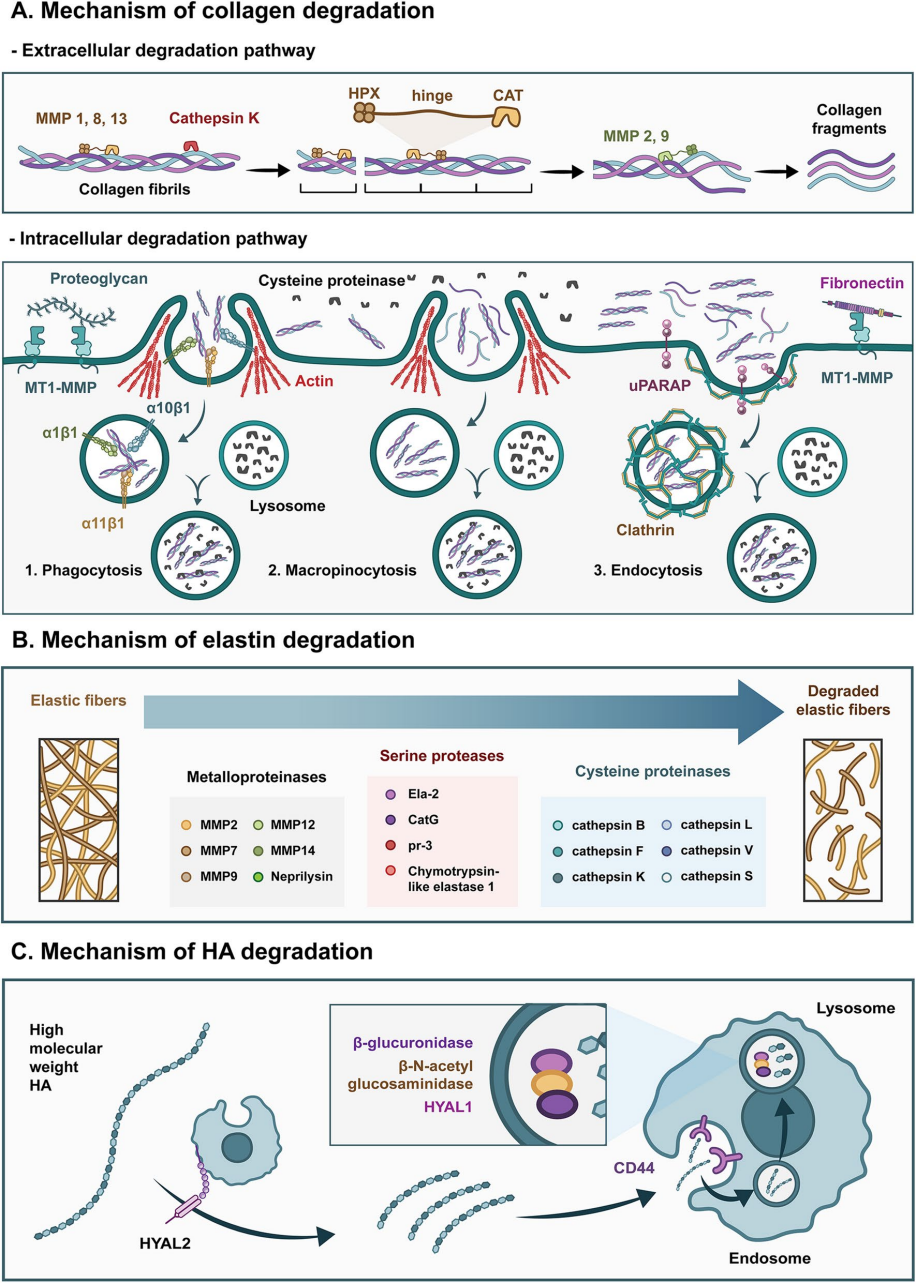
Mechanism of ECM degradation. **A** Mechanism of collagen degradation. Extracellular collagen degradation pathway: The triple helix structure of the collagen fibrils is unwound by collagenases (including MMP1, MMP8, MMP13, cathepsin K, etc.). Collagen fibrils are cleaved by these collagenases and then further degraded by gelatinases (i.e., MMP2 and MMP9). Intracellular collagen degradation pathway: During phagocytosis, uptake of collagen fibrils is mediated by the β1-integrin family and the actin-rich pseudopods. Macropinocytosis involves the uptake of collagen fragments mediated by actin. In endocytosis, collagen is recognized by the uPARAP/Endo180 receptors and subsequently taken up by clathrin-mediated vesicles. The ingested collagen is further degraded by cysteine proteases in the lysosomes. **B** Mechanism of elastin degradation. Elastin can be degraded by metalloproteinases (MMP2, MMP7, MMP9, MMP12, MMP14 and neprilysin), serine proteinases (Ela-2, CatG, PR-3 and Chymotrypsin-like elastase 1), and cysteine proteinases (cathepsin B, F, K, L, V and S). **C** Mechanism of HA degradation. HA with high molecular weight is first degraded into fragments of approximately 20 kDa by HYAL2, which is then recognized by CD44 and internalized. Finally, the fragment is completely degraded by β-glucuronidase, β-N-acetyl glucosaminidase, and HYAL1 in the lysosome

Collagen is degraded through extracellular and intracellular pathways (Sprangers and Everts 2019). In the extracellular pathway, cells secrete various collagenases including MMP1, MMP8, and MMP13, to recognize and bind to specific sites on collagen fibers through the HPX-like domain and guide the CAT domain with a zinc ion toward the cleavage site of collagen (Bertini et al. 2012). Subsequently, MMPs will unwind the triple helix structure of the collagen fibrils and cleave the intact collagen fibrils (i.e., types I, II and III collagen) at specific sites, releasing one-fourth and three-fourths lengths of the fragments (Chung et al. 2004). The CAT domain of collagenase can cleave non-collagen matrices in the presence of the HPX-like domain on MMPs, while cathepsin K, without the HPX-like domain, can degrade intact fibrillar collagens, mainly types I and II collagens. It first binds to the glycosaminoglycan (GAG) covering the collagen fibers and cleaves at the ends of the collagen and multiple sites of the triple helices (Aguda et al. 2014). Collagen fragments are then further degraded by cell-secreted gelatinases (i.e., MMP2 and MMP9) and other nonspecific proteases. The intracellular pathway comprises phagocytosis, micropinocytosis, and endocytosis. Phagocytosis acts directly on relatively intact collagen fibers, while non-collagen proteins such as fibronectin and proteoglycans that cover the surface of the collagen fibers are recognized and bound by the β1-integrin family (α1β1, α10β1, α11β1) on the membrane surface. Subsequently, actin-rich pseudopods engulf partial collagen fibers, and MT1-MMP on the membrane surface cleaves and internalizes them (Lee et al. 2007; Takaki et al. 2006). Finally, collagen is degraded by intracellular lysosomal cysteine proteases (Arora et al. 2000). Actin-mediated macropinocytosis involves the uptake of large numbers of soluble molecules, including collagen fragments, which are eventually degraded by intracellular lysosomal cysteine proteases. Macropinocytosis is the major route of collagen internalization, performing a relatively rapid and non-selective degradation of tissue. Endocytosis also acts on collagen fragments, where collagen sites bind to uPARAP/Endo180 receptors on the cell surface, followed by the formation of clathrin-mediated vesicles (Madsen et al. 2012). After fusion with lysosomes, collagen fragments are further degraded by cysteine proteases. While the extracellular degradation pathway often occurs rapidly in the pathological environment, the intracellular degradation pathway occurs relatively slowly in the physiological environment. Collectively, the extracellular and intracellular degradation pathways work in concert with each other to remodel and repair tissues concomitantly (Fig. 2A).

Insoluble elastin can be cleaved by elastases, which have been identified in the families of metalloproteinases, serine proteinases, and cysteine proteinases (Heinz 2020). Metalloproteinases with elastolytic activity include MMP2 (Diaz-Canestro et al. 2022), MMP7, MMP9, MMP12 (Mora Huertas et al. 2018), MMP14 (Miekus et al. 2019), and neprilysin (Mora Huertas et al. 2018). Four serine proteases are efficient elastases: neutrophil elastase (Ela-2), cathepsin G (CatG), protease-3 (pr-3) (Heinz et al. 2012) and Chymotrypsin-like elastase 1 (Joshi et al. 2018). Ela-2, pr-3, and CatG have been shown to completely degrade tropoelastin (Maurice et al. 2013). Six cysteine proteases have been reported to degrade elastin: cathepsin B, F (Yasuda et al. 2004), K (Panwar et al. 2020), L (Biniossek et al. 2011), V, and S (Panwar et al. 2020). The cleavage sites of elastases are mainly hydrophobic, such as Pro, Gly, Ile, Val, and Leu, or aromatic residues, such as Phe and Tyr, with some differences between elastases (Heinz 2020). For example, MMP7 showed a strong preference for Leu at P1’ (Heinz et al. 2011), while cathepsin K showed a higher affinity for Gly (Panwar et al. 2020) (Fig. 2B).

Hyaluronic acid (HA) is degraded under physiological conditions by members of the hyaluronidase (HYAL) family. Six hyaluronidases have been identified in humans, including HYAL1-4, PH-20, and HYALP1 (Papakonstantinou et al. 2012). HYAL 1 and 2 are the main hyaluronic enzymes involved in the catabolism of HA (Kaul et al. 2021). HYAL2, a glycosylphosphatidylinositol (GPI)-anchored membrane protein, hydrolyzes HA with high molecular weight and produces HA fragments of approximately 20 kDa. These fragments are then further degraded into small oligosaccharides by PH-20 (Harada and Takahashi 2007). With the recognition of HA receptors (such as CD44), HA fragments are ingested into cells and further degraded into low-molecular-weight oligosaccharides by β-glucuronidase, β-N-acetyl glucosaminidase, and HYAL1 in the lysosome (Csoka et al. 2001). Besides HYAL, hyaladherin KIAA1199 (Yoshida et al. 2013) and the transmembrane protein TMEM2 (Yamaguchi et al. 2019) show catalytic activities to degrade HA as well. Additionally, HA can be degraded through free-radical mechanisms without enzymes in the presence of molecular oxygen and reducing agents such as ascorbic acid, and cupric or ferric ions (Andre and Villain 2017) (Fig. 2C).

### Cells with degradation ability

A myriad of cells can degrade the ECM, mainly including fibroblasts, endothelial cells, macrophages, neutrophils and tumor cells (Xi and Khalil 2017)*.*

For fibroblasts, transforming growth factor (TGF)-β1 or tumor necrosis factor (TNF)-α induction can enhance the expression of MMP2 and MMP9 in fibroblasts (Kobayashi et al. 2003), while the endogenous MMP2 and MMP9 contribute to the survival and proliferation of rheumatoid arthritis (RA) synovial fibroblasts (Xue et al. 2014). Farideh Sabeh et al*.* found that MMP14 (MT1-MMP), but not soluble MMPs, was necessary for fibroblast invasion (Sabeh et al. 2009). Besides, MMP14 also played an important role in maintaining the homeostasis of the skin ECM, given that the knockout of MMP14 in mouse dermal fibroblasts resulted in impaired resolution of skin fibrosis (Zigrino et al. 2016). Wenyue Zhang et al*.* showed that fibroblast-derived MMP2 and MT1-MMP, which were involved in cervical squamous cell carcinoma (HNSCC), were critical for tumor growth and invasion (Zhang et al. 2006). Cathepsin K is synthesized by synovial fibroblasts that may be involved in collagen degradation in the bone tissue (Silva et al. 2020).

For endothelial cells, ECM remodeling is important for angiogenesis and endothelial cell tube formation. Dora Cavallo-Medved et al*.* observed that human umbilical vein endothelial cells (HUVECs) performed degradation functions by secreting active cathepsin B during an in vitro angiogenesis experiment through live cell imaging technology, accompanied by high expression of MMP2, MMP14, pro-urokinase (Pro-uPA) and urokinase-type plasminogen activator receptor (uPAR) (Cavallo-Medved et al. 2009)*.* MT1-MMP, MMP2, and MMP9 expressed by endothelial cells are involved in the process of sprouting and angiogenesis (van Hinsbergh and Koolwijk 2008)*.* Besides, they cooperate to degrade the basement membrane (i.e., type-IV collagen) and facilitate the migration of endothelial cells. Compared with MMP2 and MMP9, MT1-MMP on the surface of the cell membrane is the key nexus for endothelial cell invasion and migration (Chun et al. 2004). Studies showed that the knockout of *Mmp14* in endothelial cells in vivo affected melanoma growth and metastasis (Kümper et al. 2022). Vascular endothelial cells play a significant role in regulating the degradation of ECM by plasmin, which is essential for the development of angiogenesis (Wileman et al. 2000).

Macrophages and neutrophils are vital regulators in tissue remodeling. Briefly, the adhesion of macrophages is closely related to the protein degradation of the ECM in vitro. Macrophages-derived MT1-MMPs are critical for ECM degradation, while their surface localization is associated with macrophage migration (Linder and Scita 2015). Particularly, researchers have envisaged the effects of MMP-producing macrophages on ECM remodeling. They found that M2 macrophages and CX3CR1-positive macrophages can internalize and remove dermal collagen, thus allowing the ECM to be remodeled by fibroblasts (Madsen et al. 2013). Besides, the study investigated the behavior of macrophages on hydrogels with different densities. In dense collagen hydrogels, fibroblasts first secreted MMPs to degrade the surrounding ECM, while macrophages migrated along the path of fibroblasts and secreted MMPs continuously. In the loose collagen hydrogel network, fibroblasts initially pulled the collagen fibers into a straight line, followed by the migration of macrophages along the collagen fibers (Ford et al. 2019)*.* Furthermore, macrophages have a critical role in the process of liver fibrosis. In the initiation and progression stages, Ly6C^hi^ monocyte-derived macrophages produce TGF-β and platelet derived growth factor (PDGF), which stimulate fibroblasts differentiation into myofibroblasts and enhance ECM deposition. However, during the liver fibrosis resolution stage, their function shifts (Tacke and Zimmermann 2014). Moreover, CD11B^hi^ F4/80^int^ Ly6C^lo^ macrophages predominate at maximal fibrosis resolution with high expression of MMP9 and MMP12 (Ramachandran et al. 2012). As the co-culture of MSCs and macrophages could intensify the expression of MMPs in macrophages, the combination therapy is expected to promote the proliferation of hepatocytes and impede the process of liver fibrosis (Watanabe et al. 2019). In anti-LOXL2-treated mice, reparative monocyte-derived macrophages (MoMFs) secreted MMP14, which degraded the dense ECM (Klepfish et al. 2020). Ardi V et al*.* found that neutrophils infiltrated into the site of the primary tumor by releasing pre-stored MMP9, thereby promoting tumor angiogenesis (Deryugina et al. 2014). In addition, the exosomes from neutrophils could degrade normal collagen in the lungs by neutrophil elastase, laying a foundation for the construction of an in vitro model of chronic obstructive pulmonary disease (COPD) (Genschmer et al. 2019)*.*

**Fig. 3:**
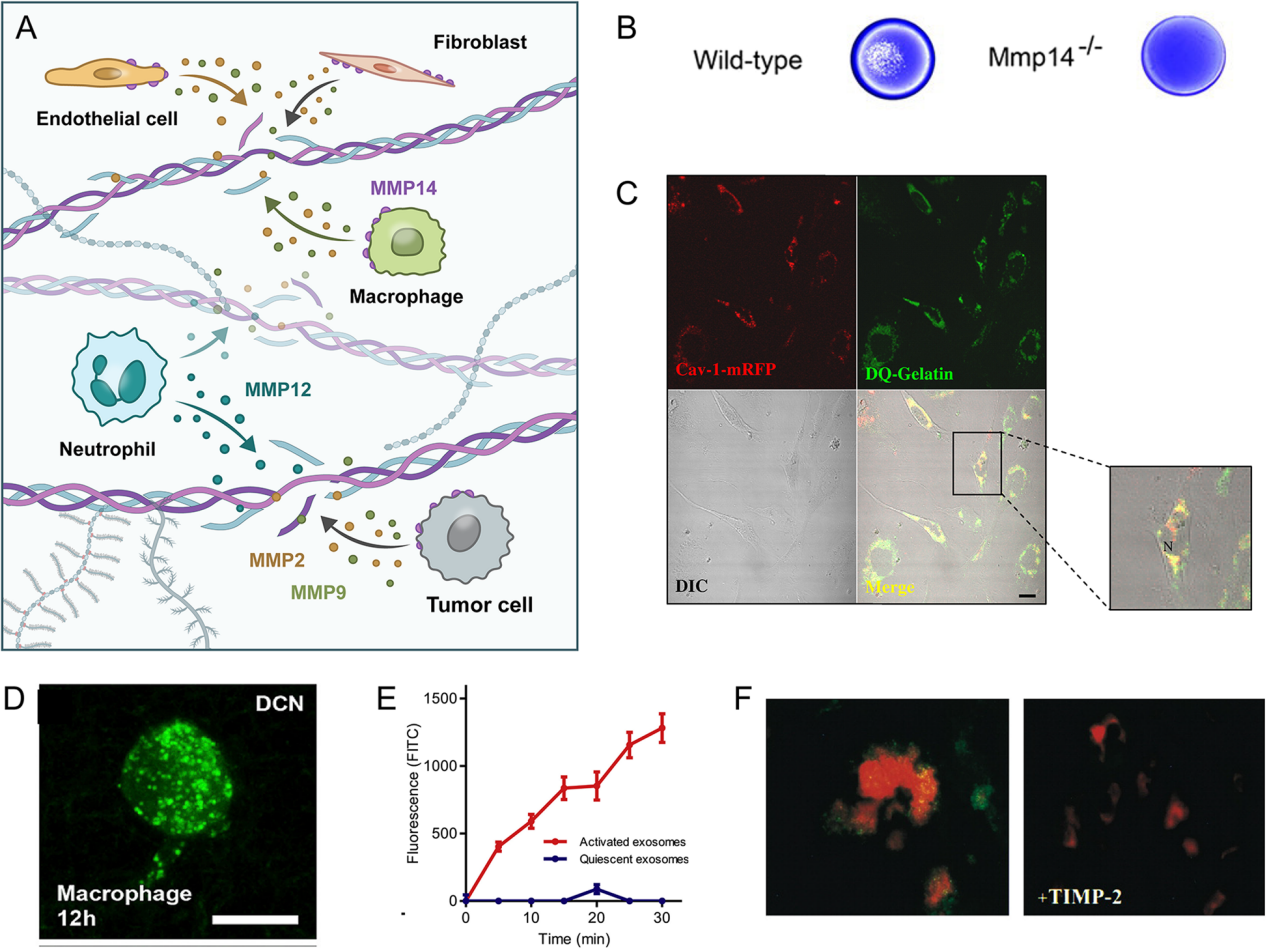
Potential cell candidates with high ECM degradation ability. **A** Schematic of cells with degradation ability, including fibroblasts, endothelial cells, macrophages, neutrophils and tumor cells. **B** Collagen degradation ability of primary murine skin fibroblasts isolated from wild-type or *Mmp14*^−/−^ mice was detected after they were cultured on type I collagen gels by Coomassie Blue staining (Sabeh et al. 2009). **C** Collagenolytic activity of human umbilical vein endothelial cells (HUVECs). HUVECs transfected with cav-1-mRFP were grown on a reconstituted basement membrane containing fluorescein conjugated-collagen IV. Confocal images were taken of cav-1-mRFP (red), substrate degradation products (green) and live cells (DIC) (Cavallo-Medved et al. 2009). Scale bars, 20 μm. **D** Endocytosis of fluorescein conjugated-collagen (green) by RAW 264.7 macrophages (Ford et al. 2019). Scale bars, 20 μm. **E** Collagenolytic activity of exosomes from activated or quiescent polymorphonuclear leukocytes was measured by culturing them with FITC-labeled type I collagen (Genschmer et al. 2019). **F** Collagen degradation ability of squamous carcinoma cells (SCC1) cultured with or without TIMP2. SCC1 cells (red) were labeled with propidium iodide and degraded collagen (green) was stained with mAb HUI77 (Hotary et al. 2003). **B**–**F** is reused with permission from Elsevier

Tumor cells are found to secrete MMPs, which assist in breaking down the dense ECM and facilitate their infiltration into other organs. Tumor cells cleave type I collagen and fibrin through MT1-MMP to invade surrounding tissues, while some other soluble MMPs have minimal effect on the degradation of the ECM (Holmbeck et al. 2003). MT1-MMP not only participates in matrix remodeling, but also activates soluble MMPs (e.g., MMP2) (van Hinsbergh and Koolwijk 2008), clearing the complement components C3b and C4b to help tumors escape from the immune system (Rozanov et al. 2004) (Fig. 3).

### Methods to characterize ECM degradation

**Fig. 4:**
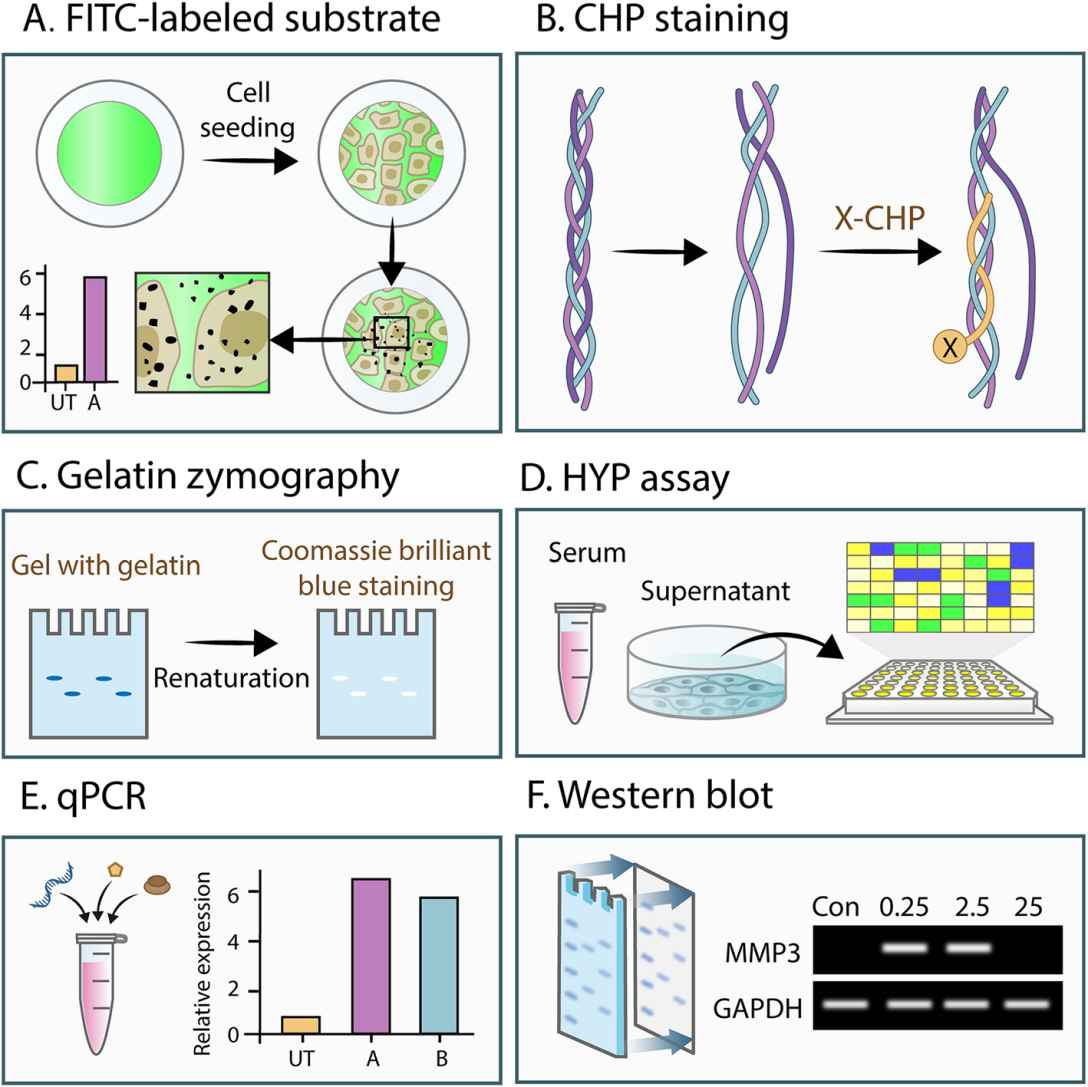
Methods to characterize ECM degradation ability of cells. **A** FITC-labeled substrate. The substrates are pre-labeled with fluorescent molecules (such as FITC) and then co-cultured with cells. Detection of areas without fluorescence can indirectly characterize the degradation ability of cells. **B** CHP staining. CHP can specifically bind denatured collagen and the degraded collagen can be stained with CHP conjugated to fluorescent molecules. **C** Gelatin zymography. Protein samples prepared from cells are separated by electrophoresis in a gel containing gelatin, and then MMPs in the samples degrade the gelatin at their respective sites, which are characterized by Coomassie Brilliant Blue staining. **D** HYP assay. The major proteins containing HYP are collagens. The HYP content of the supernatant, serum, or matrix co-cultured with cells can reflect the degree of degradation of the ECM. **E** qPCR. Real-time quantitative PCR (qPCR) can detect the expression of ECM-degrading related enzymes at the mRNA level, indicating the potential of cells to degrade the ECM. **F** Western blot. Using the specific combination of antigen and antibody, western blot can relatively qualitatively reflect the expression of ECM-degrading related enzymes at the protein level

Collagen Hybridizing Peptide (CHP) is a synthetic peptide equivalent to the collagen. It can bind specifically to denatured collagen in the form of hydrogen bonding, but is unable to bind intact collagen fibers both in vitro and in vivo. Therefore, when fluorescent-dye conjugated CHP is incubated with denatured collagen, areas of ECM degradation could be observed and quantified (Hwang et al. 2017; Li et al. 2012; Zitnay et al. 2017). Given that multiple cancer cell lines could degrade ECM at focal adhesions associated with MT1-MMP, this method can be employed to study the ECM-degrading ability of tumor cells by analyzing the irregular and dark pattern of degradation (Wang and McNiven 2012). However, the aforementioned methods are image-based and intended to analyze the ECM-degrading ability of cells qualitatively. Despite these image-based methods, the ECM-degrading ability of cells can be examined at the gene level by detecting the expression of MMP genes through qPCR (Christina et al. 2021; Moammeri et al. 2022; Popov et al. 2006). However, a high expression of MMP genes does not necessarily depict a high expression of proteins. Apart from gene detection, the ECM-degrading ability of cells can be detected through protein aspects. Hydroxyproline (HYP), which is abundant in collagen, is considered a good indicator for quantifying the levels of ECM degradation of cells (Langrock and Hoffmann 2019; Zhao et al. 2022). However, HYP is not the end product of collagen degradation and is inadequate to directly describe the ECM-degrading ability of cells (Islam et al. 2016). Besides, western blot can also quantify the expression of a certain MMP from the protein level (Kim et al. 2022; Tang et al. 2021). Nevertheless, the high expression of MMPs does not indicate a high activity of them. Gelatin zymography was used to demonstrate the activation of tumor-secreted proMMP9 by the MT1-MMP/MMP2 axis, thus hydrolyzing the surrounding ECM (Li et al. 2017; Lopez Lobato et al. 2022; Rajkumar and Mariswamy 2021). However, this method is limited to the detection of MMP2 and MMP9, and is insufficient for detecting other MMPs. In addition, despite its inefficiency in performing high-throughput screening, the commercially available collagenase kit can also be used to detect elastase on the surface of activated neutrophil-derived exosomes (Genschmer et al. 2019). Therefore, a high-throughput, inexpensive and universal method for the quantitative analysis of collagen degradation has been underwhelming thus far (Fig. 4).

## ECM-degrading cells in anti-fibrosis treatment

Deposited ECM is often overlooked in traditional fibrosis therapies. Existing studies usually focus on promoting the proliferation of tissue cells or inhibiting the activation of fibroblasts. The obstruction of tissue repair by deposited ECM needs to be taken into account in anti-fibrosis therapy. ECM-degrading cells have become therapeutics against the deposited ECM, releasing proteases (MMPs, etc.) continuously to degrade the ECM after reaching the fibrotic organ and promote the recovery of fibrosis.

In existing studies on anti-fibrosis treatment related to ECM-degrading cells, the delivered cells express proteases or inhibit the imbalance of MMPs and TIMPs in fibrotic organs (Table 1). In particular, mesenchymal stem cells (MSCs) have been reported for therapeutic applications in a variety of fibrotic organs. Sing Wan Wong et al*.* found that a soft matrix enhanced MMP production in TNF-α-stimulated MSCs, and an alginate-RGD gel-coated MSCs promoted normal ECM remodeling in the bleomycin-induced lung injury model (Wong et al. 2022). The combination of MSCs and serelaxin enhanced the activity of MMP2 in the obstructed kidney and alleviated established renal fibrosis in mice with unilateral ureteral obstruction (Huuskes et al. 2015). Additionally, human bone marrow MSCs could express MMPs in the fibrotic liver of rats induced by carbon tetrachloride (CCl_4_) and significantly decreased fibrosis (Huuskes et al. 2015). Human adipose MSCs could increase the MMP1 / TIMP1 ratio in tissues and efficiently reduced fibrosis in hypochlorite (HOCl) -induced systemic sclerosis (Maria et al. 2016). In cardiac fibroblasts cultured with MSC-conditioned medium in vitro, the expressions of α-smooth muscle actin (α-SMA) and TIMP2 were downregulated while the expression of MT1-MMP and the activities of MMP2 and MMP9 were increased. MSC transplantation correspondingly reduced cardiac ventricular fibrosis after myocardial infarction induced by coronary artery ligation (Mias et al. 2009).

**Table 1:** Studies on anti-fibrosis treatment related to ECM-degrading cells

Cell	Treatment	Mechanism	Application	Dose	Animal model	Injection method	Ref
Liver sinusoidal endothelial cells	Treatment with PMA and accutase	Increased expression of MMP1, MMP2, MMP9, etc	Advanced liver fibrosis	4 × 10^5^	6-week-old nude mice, CCl_4_ induced liver fibrosis	Intrasplenic injection	Zhao et al. 2022
MSCs	Treatment with TNF-α	Increased expression of *Mmp13*	Pulmonary fibrosis	1 × 10^5^	8 to 12-week-old C57BL/6 J mice, bleomycin-induced lung injury	Intratracheal injection	Wong et al. 2022
Bone MSCs	Overexpression of Smad7	Increased expression of MMP1	Liver cirrhosis	(3~5) × 10^6^	6-week-old Wistar rats, CCl_4_ induced liver fibrosis	Intrahepatic injection	Su et al. 2020
Clonal mesenchymal stem cells	-	Increased expression of MMP2 and MMP9	Liver fibrosis	3 × 10^7^	6-week-old Wistar rats, CCl_4_ induced liver fibrosis	Intrasplenic injection	Hardjo et al. 2009
MSCs	Combination therapy with serelaxin	Increased expression of MMP2 in kidney	Renal fibrosis	1 × 10^6^	Male C57BL/6 J mice, unilateral ureteral obstruction induced renal fibrosis	Renal vein injection	Huuskes et al. 2015
Bone marrow mesenchymal stromal cells	-	Increased expression of MMP13 in M2 macrophage	Liver fibrosis	5 × 10^5^	10-week-old C57BL/6 J male mice, CCl_4_ induced liver fibrosis	Tail vein injection	Luo et al. 2019
Human amnion epithelial cells	-	Downregulation of *TIMP1*, *2*, *3*, *4* in lung	Pulmonary fibrosis	1 × 10^6^	8-week-old SCID mice, bleomycin induced pulmonary fibrosis	Tail vein injection	Moodley et al. 2010
Adipose-derived stem cells		Increased expression of MMP9 in liver	Liver fibrosis	1 × 10^6^	8-week-old male Wistar rats, TAA induced liver fibrosis	Intrahepatic injection	Harn et al. 2012
Kupffer cells	-	Increased expression of MMP9	Liver fibrosis	2 × 10^6^	C57BL/6 mice, TAA induced liver fibrosis	Intravenous injection	Feng et al. 2018
MSCs	Overexpression of hepatocyte growth factor (HGF)	Increased expression of *MMP9*, *13*, *14* and uPA, decreased expression of *TIMP1* in liver	Liver fibrosis	1 × 10^7^	5-week-old Sprague–Dawley (SD) male rats, DMN induced liver fibrosis	Intrasplenic injection	Kim et al. 2014
Bone marrow-derived liver stem cells	Overexpression of uPA	Activation of pro-MMP3 by uPA, which activates MMP2 and MMP9, then degrading collagen	Liver fibrosis	2 × 10^6^	Female F344 rats, CCl_4_ induced liver fibrosis	Tail vein injection	Sun et al. 2008
Bone marrow-derived MSCs	Treatment with IC-2	Increased expression of MMP1, MMP2, and MMP14	Liver fibrosis	-	7–9-week-old BALB/c-nu/nu male mice, CCl_4_ induced liver fibrosis	Intrahepatic transplantation	Itaba et al. 2019
Bone marrow‑derived MSCs	Transfection with survivin	Increased expression of *MMP9* in lung	Pulmonary fibrosis	1 × 10^6^	6–8-week-old C57BL/6 male mice, bleomycin induced pulmonary fibrosis	Caudal vein injection	Zhou et al. 2015
Induced pluripotent stem cells	-	Inhibition of imbalance in the expression ratios of MMP2/TIMP2 and MMP9/TIMP1	Pulmonary fibrosis	2 × 10^6^	C57BL/6 male mice, bleomycin-induced pulmonary fibrosis	Intravenous injection	Zhou et al. 2016
Bone marrow MSCs	-	Increased expression of MMPs	Liver fibrosis	1 × 10^6^	Adult male Wistar Kyoto (WKY) rats, CCl_4_ induced liver fibrosis	Portal vein injection	Zhao et al. 2012
Endometrial regenerative cells	-	Increased expression of *MMP9*	Pulmonary fibrosis	1 × 10^6^	6–8-week-old C57BL/6 female mice, bleomycin-induced pulmonary fibrosis	Tail vein injection	Zhao et al. 2018
Bone-marrow-derived macrophages	Treatment with lipopolysaccharide (M1-polarized)	Increased expression of *MMP2*, *9*, and *13* in recruited Ly6C^lo^ macrophages	Liver fibrosis	1 × 10^6^	8-week-old C57BL/6 male mice, CCl_4_ induced liver fibrosis	Tail vein injection	Ma et al. 2017
Bone marrow cells	-	Increased expression of MMP13 and MMP9 in some of bone marrow-derived cells and liver resident cells	Liver fibrosis	5 × 10^6^	6-week-old C57BL/6 mice, CCl_4_ induced liver fibrosis	Intravenous injection	Higashiyama et al. 2007
Bone marrow-derived MSCs	-	Increased expression of *MMP2* in liver	Liver fibrosis	3 × 10^6^	Male Wistar rats, BDL induced liver fibrosis	Tail vein injection	Mohamed et al. 2016
Chorionic plate-derived MSCs	-	Increased expression of MMP9 in liver	Liver fibrosis	2 × 10^6^	6-week-old male Sprague–Dawley rats, CCl_4_ induced liver fibrosis	Intrahepatic transplantation	Lee et al. 2010
Adipose-derived MSCs	-	Enhanced ratio of *Mmp1*/*Timp1* in skin and lung tissues	Systemic sclerosis	2.5 × 10^5^	C57BL/6 mice, hypochlorite (HOCl) induced systemic sclerosis	Tail vein injection	Maria et al. 2016
Bone marrow-derived MSCs	-	Enhanced ratio of MMP/TIMP production by cardiac fibroblasts	Cardiac ventricular fibrosis	3 × 10^6^	Lewis congenic rats, interventricular artery ligation induced myocardial infarction	Intramyocardial injection	Mias et al. 2009

At present, there is a lack of research on anti-fibrosis treatment through cell-mediated ECM degradation. Most notably, Zhao et al. found that liver sinusoidal endothelial cells stimulated by accutase and phorbol myristate acetate (PMA) showed significant ECM degradation and relieved CCl_4_-induced advanced liver fibrosis in mice (Zhao et al. 2022). In addition, stimulated HUVECs also showed the ability to degrade the ECM and treat liver fibrosis, demonstrating their potential for clinical application.

## Conclusions and perspectives

### Conclusion

The degradation of the ECM is essential for the reversal of fibrosis, and a variety of proteases are involved in this process. Several types of cells can degrade ECM through extracellular or intracellular pathways in certain physiological states. The ECM-degrading ability of cells can be characterized by detecting the expression of related enzymes or analyzing co-cultured substrates. ECM-degrading cells are expected to degrade the deposited ECM in fibrotic organs to treat fibrosis, and the feasibility of this therapeutic strategy has been demonstrated in several studies.

### Potential causes of limiting the clinical application of ECM-degrading cells in anti-fibrosis treatment

By secreting MMPs, ECM-degrading cells can degrade the ECM, potentially providing a therapeutic benefit to fibrotic diseases. Nevertheless, multiple factors impede the application of ECM-degrading cells in clinical settings for anti-fibrosis treatment.

First, when liver fibrosis progresses to cirrhosis, the normal structure of the liver is severely disrupted with excessive fibronectin and collagens I, III, V, and VI deposited in the fibrotic septa (Iredale et al. 2013). Additionally, the biophysical properties of the ECM are changed by cross-linking reactions. Most collagen cross-linking is mediated by lysyl oxidases (LOX), transglutaminases (TGase), and advanced glycosylation end products (AGEs), which increase the difficulty of degrading the ECM that cannot be easily broken down (Kong et al. 2021; Lyu et al. 2023). ECM-degrading cells may not possess the capability to break down the cross-linked and multi-component ECM in vivo. Therefore, we should devise an efficient screening system to detect the ECM-degrading potential of cells in vitro, taking the bionics of the ECM components and the degree of cross-linking into account.

Second, previous studies demonstrated that ECM-degrading liver sinusoidal endothelial cells possessed a strong ECM degradation ability for liver fibrosis (Zhao et al. 2022); however, there is insufficient evidence of ECM-degrading cells with similar capability for other fibrotic diseases, such as lung fibrosis, skin fibrosis, and myocardial fibrosis. In the future, we can use single-cell sequencing data from fibrotic and normal organs to determine the cell types with high expression of ECM-degrading proteases; these cells may serve as potential treatments for fibrosis.

Third, the safety of cell therapies in clinical application is an unavoidable issue, as allogeneic cells may cause immune responses in patients. Besides safety, precise delivery of therapeutic cells to the liver fibrotic areas in vivo should be taken into account to preclude damages caused to other organs. How to avoid degradation in healthy tissues and excessive degradation in fibrotic organs needs to be considered. In addition, for organs with weak regeneration capabilities, it is uncertain whether the structure and function of fibrotic tissues can be restored after ECM degradation.

Fourth, human-derived cell therapies may be clinically viable; yet, the majority of studies have only utilized nude mice to assess the efficacy of anti-fibrosis treatments with ECM-degrading cells (Cao et al. 2017; Nakamura et al. 2016; Nakamura et al. 2012; Woo et al. 2012). To further explore the effects of ECM-degrading cells, immunocompetent mice and primates should be employed in the future. Moreover, multiple causative models of fibrosis should be taken into account.

Fifth, a pro-inflammatory microenvironment, an imbalance of MMPs and TIMPs, and necrosis of certain cells can all be causes of fibrotic diseases (Tan et al. 2021). ECM-degrading cells may primarily be responsible for degrading the ECM that has been deposited. Currently, increasing clinical trials based on cell therapies, especially MSC therapy, for treating a myriad of diseases have achieved unprecedented breakthroughs. MSCs can be utilized to regulate the immune microenvironment and further augment the proliferation of particular cells (Nakamura et al. 2012). In the future, we can develop a combination of ECM-degrading cells and MSC therapies to facilitate organ regeneration while simultaneously degrading scars.

### ECM-degrading cells derived vesicles

Despite the cells, cell-derived vesicles could release MMPs to promote tumor invasion (Turturici et al. 2014). Therefore, there is the possibility that cell-derived vesicles can degrade the ECM through the secretion and delivery of metalloproteinases to degrade the deposited ECM in the fibrotic area, thus promoting the recovery of fibrosis. Compared with ECM-degrading cell therapy, ECM-degrading vesicle therapy will have numerous advantages. Briefly, cell-derived vesicles are less immunogenic and more persistent in the circulatory system (Kazemi and Sobhania 2018). Furthermore, cell-derived vesicles are smaller in size, which will benefit their infiltration into the densely structured fibrotic tissue to perform degradation functions. However, at present, due to a lack of cell-derived vesicles with high degradability and low vesicle yields (Yamashita et al. 2018), the clinical application of cell-derived vesicles for fibrosis treatment has been limited.

First, it is challenging and arduous to screen cells with high degradation ability due to the low throughput and cost-ineffective methods as mentioned above. Therefore, it is a prerequisite to develop a platform for high-throughput and quantitative detection of the cellular degradation capacity.

Besides, during the production of cell-derived vesicles, traditional methods for isolating native extracellular vesicles are used, including ultracentrifugation, size- exclusion chromatography, and immunocapture. However, regardless of the isolation methods, the amount of vesicle protein obtained was low and insufficient for downstream applications (Coumans et al. 2017). In recent years, the method of culturing cells on a large-scale bioreactor, collecting the supernatant, and isolating extracellular vesicles has gained much interest. This method greatly liberates manpower and is cost-effective. However, the complicated methods of application have hindered their utilization, indicating that the culture device requires constant adjustment of the environmental parameters promptly to prevent high shear stress which may cause cell death (Colao et al. 2018). Therefore, great strides have been made by scientists to produce engineered vesicles which are reassembled by cell membranes. Using a liposome extrusion preparation device, a study sequentially passed the U937 cells and Raw264.7 cells through filter membranes with pore sizes of 10 μm, 5 μm, and 1 μm. Then, these cells were broken into nanovesicles, which have physicochemical and biological properties similar to those of exosomes (Jang et al. 2013). However, this method relies on manpower, which is relatively uncontrollable. Therefore, the scientists hope that automated extrusion control can be performed through microfluidics. Yong Song Gho et al. proposed a novel and efficient method for ESCs to enter narrow, hydrophilic microchannels, rupture their membranes, and generate artificial nanovesicles. These nanovesicles contained mRNA, intracellular proteins, and plasma membrane proteins, and they had a similar shape to exosomes secreted by cells (Jo et al. 2014). However, studies of producing vesicles using microchannels are limited to ESCs, and the confined dimension of the channel (5 μm) results in poor vesicle morphology and uneven particle size. In conclusion, the discovery of the ECM-degrading ability of various cells has paved the way for the development of effective new treatments for fibrotic diseases. The development of a universal preparation process suitable for a variety of cell types is necessary to engender an easy-to-operate, controllable preparation process to obtain nanovesicles with good quality and high yield.

## Data Availability

Not applicable.

## Abbreviations

AGE: Advanced glycosylation end products
CAT: Catalytic
CatG: Cathepsin G
CCl_4_: Carbon tetrachloride
CHP: Collagen Hybridizing peptide
COPD: Chronic obstructive pulmonary disease
ECM: Extracellular matrix
Ela-2: Neutrophil elastase
GAG: Glycosaminoglycan
GPI: Glycosylphosphatidylinositol
HA: Hyaluronic acid
HNSCC: Cervical squamous cell carcinoma
HOCl: Hypochlorite
HPX: Hemopexin
HUVEC: Human umbilical vein endothelial cell
HYAL: Hyaluronidase
HYP: Hydroxyproline
LOX: Lysyl oxidases
MMP: Matrix metalloproteinase
MoMF: Monocyte-derived macrophage
MSC: Mesenchymal stem cell
MT-MMP: Membrane-type matrix metalloproteinase
PA: Plasminogen activator
PDGF: Platelet derived growth factor
PMA: Phorbol myristate acetate
pr-3: Protease-3
pro-uPA: Pro-urokinase-type plasminogen activator
qPCR: Real-time quantitative PCR
SCC1: Squamous carcinoma cell
SMA: Smooth muscle actin
TGase: Transglutaminases
TGF: Transforming growth factor
TIMP: Tissue inhibitors of metalloproteinase
TM: Transmembrane
TNF: Tumor necrosis factor
tPA: Tissue plasminogen activator
uPA: Urokinase plasminogen activator
uPAR: Urokinase-type plasminogen activator receptor
